# Comparative Efficacy and Safety of Tissue Grafting Versus ReCell Autologous Cell Suspension in Stable Vitiligo: A 12‐Month Retrospective Study

**DOI:** 10.1111/jocd.71086

**Published:** 2026-07-27

**Authors:** Dihui Lai, Jianchun Hao, Xueya Tong, Maoying Wei, Xiaoju Xian, Xintong Chang, Jing Ma, Shaowei Cheng

**Affiliations:** ^1^ Department of Dermatology Chui Yang Liu Hospital Affiliated to Tsinghua University Beijing China; ^2^ Department of Dermatology Chengdu Borun Vitiligo Hospital Chengdu China

**Keywords:** phototherapy, ReCell, retrospective study, split‐thickness skin grafting, suction blister epidermal grafting, vitiligo surgery

## Abstract

**Background:**

Vitiligo surgery is often the last resort for treatment‐resistant lesions with stable conditions. Between tissue grafting (suction blister epidermal grafting [SBEG] and ultra‐thin split‐thickness skin grafting [UT‐STSG]) and cellular grafting (ReCell), large‐scale comparative data are lacking.

**Methods:**

We conducted a comprehensive retrospective cohort study of 277 patients (1501 lesions) undergoing tissue grafting (*n* = 111, 451 lesions), or ReCell (*n* = 166, 1050 lesions) for stable vitiligo between December 2023 and March 2026. The primary endpoint was 12‐month repigmentation rate, with secondary outcomes including adverse events. Predictors of treatment success were identified via binary logistic regression.

**Results:**

At 12 months, ReCell achieved superior repigmentation compared with tissue grafting (median 89.7% vs. 60.1%, *p* < 0.001), with ≥ 75% repigmentation in 62.8% versus 24.0% of lesions. Stratified analysis revealed site‐dependent effects: no significant difference was observed for facial/trunk lesions (98.6% vs. 92.9%, *p* = 0.562) or acral lesions (46.9% vs. 50.8%, *p* = 0.077), whereas ReCell was superior for peri‐mucosal sites (81.0% vs. 50.0%, *p* = 0.028). In nonsegmental vitiligo, ReCell outperformed tissue grafting (87.5% vs. 53.9%, *p* < 0.001), with no difference in segmental disease (95.0% vs. 81.3%, *p* = 0.374). Adjuvant 308‐nm excimer lamp therapy (24–47 sessions) increased success fourfold (OR = 3.90, *p* = 0.002). ReCell demonstrated superior safety (2.4% vs. 16.2% adverse events, *p* = 0.001).

**Conclusion:**

ReCell may offer superior 12‐month repigmentation and safety for stable nonsegmental, non‐acral vitiligo. Traditional tissue grafting retains viability for limited lesions and resource‐limited settings. Postoperative phototherapy is crucial for optimal outcomes. These real‐world data are hypothesis‐generating; prospective randomized trials with extended follow‐up are needed.

## 1 Introduction

Vitiligo is a common acquired depigmenting skin disorder affecting approximately 0.5%–2% of the global population [1]. Due to its disfiguring characteristics, it can severely impact patients' psychosocial well‐being [2, 3].

Based on the clinical skin manifestations and Wood's lamp examination results, vitiligo disease activity can be classified into the progressive phase and stable phase [1]. Therapeutic strategies for vitiligo are phase‐dependent. For progressive vitiligo, oral and topical agents along with phototherapy remain mainstay treatments. However, for stable vitiligo, some patients exhibit poor responses to medications or phototherapy, and the remaining refractory lesions often pose a clinical challenge. In such circumstances, surgical intervention emerges as a viable therapeutic alternative, specifically indicated for refractory lesions that have demonstrated stability for a minimum of 12 months [1].

Depending on donor harvesting techniques, surgical interventions can be categorized into tissue grafting and cellular grafting methods [4]. Tissue grafting encompasses three primary modalities: suction blister epidermal grafting (SBEG), split‐thickness skin grafting (STSG), and mini‐punch grafts. SBEG, first introduced by Kiistala and Mustakallio [5], remains the most widely employed technique for vitiligo surgery due to its technical simplicity and favorable cost‐effectiveness [6, 7]. However, it also has drawbacks such as being time‐consuming, causing discomfort at the donor site during blister suction, and carrying a risk of variegated appearance. STSG, initially described in the early 1960s, represents another tissue‐based approach for vitiligo surgery [8]. This technique can be subclassified by graft thickness into ultra‐thin, thin (Thiersch–Ollier), intermediate (Blair–Brown), and thick (Padgett) variants [4]. While Kahn et al. have demonstrated the efficacy of ultra‐thin STSG in treating vitiligo [9], its clinical application is constrained by the limited donor area per harvest, rendering it unsuitable for patients with extensive disease involvement.

With technological advancement, cellular grafting has emerged to address the challenge of extensive vitiligo. The main technique used is autologous non‐cultured epidermal cell suspension (ReCell). This technique uses trypsin to lyse epidermal keratinocytes, which are then diluted at a certain ratio and applied to skin lesion areas exceeding 10 times the donor site size [10]. This approach achieves satisfactory clinical outcomes while circumventing stringent laboratory requirements, making it particularly suitable for patients with large‐area disease or anatomically complex lesions [11]. However, since reagent kits are required during the procedure, the single‐surgery operational cost cannot be ignored.

Regarding the clinical advantages and disadvantages of different surgical methods, multiple studies have conducted clinical comparisons, and some scholars have performed systematic reviews and meta‐analyses [12, 13, 14]. However, common issues include small sample sizes, insufficient evaluation of site‐specificity and synergistic effects with phototherapy, and a lack of relevant research directly comparing mainstream surgical methods. Therefore, the superior and inferior characteristics of each surgery have not been given a relatively clear conclusion. To address this, we conducted a real‐world large‐sample retrospective analysis of stable, refractory vitiligo patients who have undergone SBEG, ultra‐thin STSG, or ReCell transplantation over a certain period. We hope to directly compare the efficacy and safety profiles of these modalities and to evaluate potential interactions between lesion location, vitiligo type, and surgical outcomes.

## 2 Materials and Methods

### 2.1 Study Design and Patient Selection

This retrospective cohort study evaluated patients with stable vitiligo who underwent surgical treatment at Chengdu Borun Vitiligo Hospital between December 2023 and March 2026. The study protocol was approved by the institutional ethics committee (Approval No.: 2026BR‐01002) and conducted in accordance with the Declaration of Helsinki. Written informed consent was obtained from all patients or their legal guardians. Separate written consent was obtained for the publication of clinical photographs, with specific attention to ensuring that identifiable facial features were either excluded from publication or additionally authorized by the patient.

Patients were eligible if they had: (1) stable vitiligo for ≥ 12 months based on clinical history and Wood's lamp examination [1]; (2) undergone SBEG, ultra‐thin STSG (UT‐STSG), or autologous non‐cultured epidermal cell suspension (ReCell) transplantation; and (3) completed ≥ 12 months of postoperative follow‐up. Exclusion criteria comprised pregnancy or lactation, keloid tendency, active skin infection, coagulopathy, or severe systemic disease. Patient demographics and clinical characteristics are presented in Table 1.

**TABLE 1:** Demographic and clinical characteristics of patients.

	Tissue grafting (*n* = 111)	ReCell (*n* = 166)	Total (*n* = 277)	*p*
No. of lesions, *n* (%)	451 (30.05%)	1050 (69.95%)	1501	
Age, years, mean ± SD (range)	23.24 ± 12.47 (7–67)	20.62 ± 12.27 (7–71)	21.67 ± 12.40 (7–71)	0.086
Gender, *n* (%)				
Female	51 (45.95%)	90 (54.22%)	141 (50.90%)	0.177
Male	60 (54.05%)	76 (45.78%)	136 (49.10%)	
Fitzpatrick skin phototypes, *n* (%)				
III	99 (89.19%)	157 (94.58%)	256 (92.42%)	0.109
IV	12 (10.81%)	9 (5.42%)	21 (7.58%)	
Vitiligo type, *n* (%)				
Nonsegmental	93 (83.78%)	126 (75.90%)	219 (79.06%)	0.002
Segmental	10 (9.01%)	37 (22.29%)	47 (16.97%)	
Unclassified	8 (7.21%)	3 (1.81%)	11 (3.97%)	
Comorbidity, *n* (%)			11 (3.97%)	
Thyroid disease			2 (0.72%)	
Others such as diabetes, insomnia, epilepsy, etc.			9 (3.25%)	
Family history, *n* (%)	14 (5.05%)	13 (4.69%)	27 (9.75%)	0.188
Duration of disease, years	6.36 ± 0.45 (2, 27)	6.67 ± 0.66 (2, 36.5)	6.49 ± 0.59 (2, 36.5)	0.671
Lesion sites (total 1501), *n* (%)				
Face/neck/trunk	56 (3.73%)	717 (47.77%)	773 (51.50%)	< 0.001
Acral	356 (23.72%)	283 (18.85%)	639 (42.57%)	
Peri‐mucosal	39 (2.60%)	50 (3.33%)	89 (5.93%)	
Combined 308‐nm excimer lamp treatment				
< 24 sessions	74 (26.43%)	115 (44.64%)	189 (67.5%)	0.703
24–47 sessions	33 (11.79%)	43 (15.36%)	86 (30.71%)	
> 47 sessions	5 (1.79%)	10 (3.57%)	15 (5.36%)	

### 2.2 Surgical Procedures

All procedures were performed by experienced dermatologic surgeons using standardized protocols (detailed in Table S1).

#### 2.2.1 Tissue Grafting (SBEG and UT‐STSG)

Following local povidone–iodine disinfection and appropriate local anesthesia (1% lidocaine infiltration for SBEG; topical compound lidocaine cream for UT‐STSG donor site to ensure smooth skin surface for graft harvesting), epidermal blisters were induced using a KN‐3000 suction device (Xuzhou Kenuo Medical Instrument Equipment Co. Ltd. China) at negative pressure 300–375 mmHg and 45°C for 40–90 min (SBEG) or a 0.13‐mm skin graft was harvested from the medial thigh using a blade (UT‐STSG). Recipient sites were dermabraded to punctate (SBEG) or diffuse (UT‐STSG) bleeding using a BFY‐IVC dermabrasion machine (Shaoxing Satellite Medical Equipment Co. Ltd. China). Harvested tissues were transferred, compressed, and dressed for 7 days.

#### 2.2.2 ReCell Autologous Non‐Cultured Epidermal Cell Suspension

Split‐thickness grafts (0.15–0.2 mm) were harvested from medial thigh or perilesional skin at a 1:10 donor‐to‐recipient ratio and processed using the ReCell kit (AVITA Medical, Australia) to generate a keratinocyte–melanocyte–fibroblast suspension [15, 16]. Recipient sites were dermabraded to pinpoint bleeding, followed by uniform spray application of the cell suspension. The treated area was covered with Tegaderm I.V. transparent film (3M, USA) and sterile gauze for 7 days.

### 2.3 Outcome and Follow‐Up

The primary outcome was repigmentation rate assessed independently by two blinded dermatologists based on clinical photography at 3, 6, and 12 months postoperatively, using a validated 4‐grade scale: poor (< 25%), fair (25%–49%), good (50%–74%), and excellent (≥ 75%). Treatment success was defined as ≥ 50% repigmentation at 12 months. Secondary outcomes included adverse events recorded at each follow‐up visit.

All patients received routine postoperative traditional Chinese medicines as institutional protocol; exact composition is proprietary but uniform across groups. Additionally, some patients started adjuvant phototherapy twice weekly with 308‐nm excimer light 2 weeks post‐surgery.

### 2.4 Statistical Analysis

Data were analyzed using SPSS version 26.0 (IBM Corp., USA). Continuous variables are expressed as mean ± SD or median (interquartile range), and categorical variables as frequencies and percentages. Between‐group comparisons were performed using Mann–Whitney *U* test (for two‐group comparisons) or Kruskal–Wallis *H* test (for three‐group comparisons), Pearson *χ* ^2^ test, or Fisher's exact test, as appropriate. Binary logistic regression was used to identify independent predictors of 12‐month treatment success. Statistical significance was set at *p* < 0.05.

## 3 Results

During the study period, a total of 735 patients with vitiligo underwent these three surgical treatments at this institution. After applying the inclusion and exclusion criteria, a final total of 277 patients with 1501 lesions were included: 111 patients (451 lesions) in the tissue grafting group and 166 patients (1050 lesions) in the ReCell group. Baseline characteristics were generally comparable across groups, with the exception of lesion location (*p* < 0.001) and vitiligo type distribution (*p* = 0.002) (Table 1). Technique‐specific lesion distributions for SBEG versus UT‐STSG are presented in Table S2.

**FIGURE 1:**
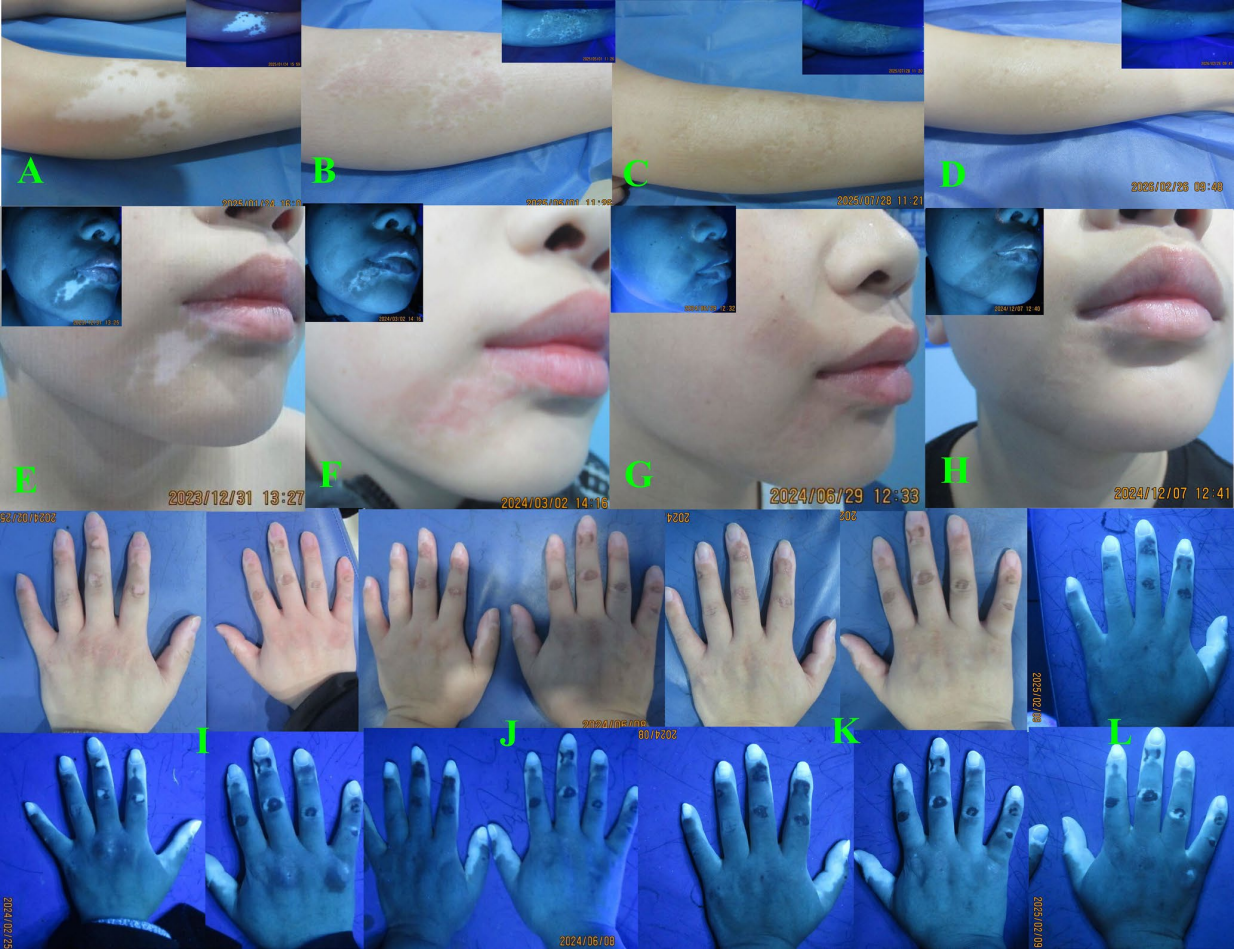
Representative clinical photographs illustrating three vitiligo surgical techniques. (A–D) A 28‐year‐old female with 5‐year history of refractory vitiligo on the right leg: (A) before ReCell transplantation; (B) 3 months postoperatively; (C) 6 months postoperatively demonstrating nearly 95% repigmentation; (D) after 12 months, the repigmentation remains stable. (E–H) A 20‐year‐old female with 10‐year history of segmental refractory vitiligo on the right mandible: (E) before SBEG; (F) 3 months postoperatively; (G) 6 months postoperatively showing near 100% repigmentation; (H) 12 months postoperatively. (I–L) A 36‐year‐old male with 15‐year history of refractory acral vitiligo: (I) before UT‐STSG; (J) 3 months postoperatively; (K) 6 months postoperatively with approximately 50% repigmentation; (L) 12 months postoperatively, the condition of repigmentation of the left hand lesion remained stable, but a small area of lesion recurrence appeared on the right hand.

At 12 months postoperatively, repigmentation rates differed significantly between the two modalities. ReCell achieved a median repigmentation rate of 90.00% (IQR 32.6%), significantly higher than tissue grafting at 60.00% (IQR 70.62%) (*p* < 0.001). The proportion of lesions achieving ≥ 75% repigmentation was similarly highest with ReCell (62.76%), followed by tissue grafting (23.95%) (*p* < 0.001). At 6 months, a parallel trend was observed (ReCell 62.29% vs. tissue grafting 22.62% achieving ≥ 75% repigmentation), confirming early separation of outcomes (Table 2, Figure 1).

**TABLE 2:** Detailed repigmentation rates (*N* = 1501 lesions) and adverse events.

Repigmentation rate	Tissue grafting, *n* (%)	ReCell, *n* (%)	*p*
3 months			
< 25	118 (26.16%)	241 (22.95%)	0.014
[25, 50)	112 (24.83%)	128 (12.19%)	
[50, 75)	135 (29.93%)	288 (27.43%)	
[75, 100]	86 (19.07%)	393 (37.43%)	
6 months			
< 25	139 (30.82%)	159 (15.14%)	< 0.001
[25, 50)	90 (19.96%)	91 (8.67%)	
[50, 75)	120 (26.61%)	146 (13.90%)	
[75, 100]	102 (22.62%)	654 (62.29%)	
12 months			
< 25	136 (30.16%)	154 (14.67%)	< 0.001
[25, 50)	93 (20.62%)	92 (8.76%)	
[50, 75)	114 (25.28%)	145 (13.81%)	
[75, 100]	108 (23.95%)	659 (62.76%)	
Adverse events, *n* (%)			
Postoperative pain	1 (0.90%)	0 (0.0%)	0.001
Hyperpigmentation	15 (13.51%)	4 (2.41%)	
Scar	1 (0.90%)	0 (0.0%)	
Milia	1 (0.90%)	0 (0.0%)	

A binary logistic regression analysis was performed on patients with effective repigmentation and ineffective repigmentation at the 12‐month follow‐up to evaluate the impact of patients' basic characteristics on the repigmentation rate. The results showed that factors such as age, gender, Fitzpatrick skin phototypes, family history, presence or absence of comorbidities, disease duration, surgical modality, and type of vitiligo had no clear influence on the repigmentation. However, lesion location, number of lesions, and treatment frequency grades of postoperative 308‐nm excimer lamp therapy on repigmentation rate were three independent predictive factors (Table 3).

**TABLE 3:** Results of binary logistic regression for treatment success.

	*B*	*p*	OR (95% CI)
Surgical modality	0.618	0.065	1.854 (0.962, 3.574)
Lesions sites	−1.111	< 0.001	0.329 (0.212, 0.511)
Acral vs. face/neck/trunk	−2.276	< 0.001	0.103 (0.043, 0.244)
Acral vs. peri‐mucosal	−0.044	0.918	0.957 (0.416, 2.200)
No. of lesions	−0.100	< 0.001	0.905 (0.858, 0.954)
Vitiligo type	0.551	0.170	1.734 (0.789, 3.810)
Phototherapy frequency	0.932	0.002	2.540 (1.401, 4.604)
24–47 sessions vs. 0–23 sessions	1.36	0.002	3.904 (1.682, 9.059)
> 47 sessions vs. 0–23 sessions	1.086	0.124	2.964 (0.743, 11.825)

Abbreviations: *B*, unstandardized coefficient; CI, confidence interval; OR, odds ratio; vs., versus.

Among them, lesion site was the strongest predictive factor (OR = 0.33, 95% CI: 0.21–0.51, *p* < 0.001); acral location conferred a 90% reduction in success odds compared with face/neck/trunk (OR = 0.10, 95% CI: 0.04–0.24, *p* < 0.001). Each additional lesion reduced success odds by approximately 10% (OR = 0.91, 95% CI: 0.86–0.95, *p* < 0.001). After adjusting for site, lesion number, type, and phototherapy, ReCell maintained a trend toward higher success compared with tissue grafting (OR = 1.85, 95% CI: 0.96–3.57, *p* = 0.065).

Postoperative 308‐nm excimer lamp therapy demonstrated a frequency‐dependent benefit. Patients receiving 24–47 sessions had a nearly fourfold higher success rate compared with those receiving < 24 sessions (OR = 3.90, 95% CI: 1.68–9.06, *p* = 0.002). The effect of > 47 sessions was directionally similar but did not reach statistical significance (OR = 2.96, 95% CI: 0.74–11.83, *p* = 0.124), limited by small sample size (*n* = 15) (Tables 1 and 3).

Furthermore, because the distribution of lesion sites and types of vitiligo was uneven at baseline between the two surgical approaches, we conducted a stratified analysis for these two factors (Table S3). By anatomical site: For face/neck/trunk lesions, tissue grafting and ReCell showed comparable high efficacy (median 92.9% vs. 98.6%, *p* = 0.562). For acral lesions, both techniques performed suboptimally, with no significant difference between groups (46.9% vs. 50.8%, *p* = 0.077), indicating that anatomical factors dominate at these refractory sites. For peri‐mucosal lesions, ReCell demonstrated significantly superior repigmentation (81.0% vs. 50.0%, *p* = 0.028). By vitiligo type: In nonsegmental vitiligo (*n* = 219), ReCell markedly outperformed tissue grafting (87.5% vs. 53.9% achieving ≥ 50% repigmentation, *p* < 0.001). In segmental vitiligo (*n* = 47), high repigmentation rates across both groups eliminated between‐group differences (95.0% vs. 81.3%, *p* = 0.374), though ReCell maintained a numerical advantage. Within the ReCell group, segmental disease trended toward superior outcomes compared with nonsegmental disease (95.0% vs. 87.5%), consistent with its inherent pathogenic stability.

Adverse events were significantly less frequent with ReCell (4 cases, 2.41%) compared with tissue grafting (18 cases, 16.21%) (*p* = 0.001). Hyperpigmentation was the most common event, occurring in 2.41% of ReCell, 13.51% of tissue grafting. Tissue grafting was additionally associated with isolated cases of postoperative pain, scarring, and milia (each < 1%) (Table 2). No donor site complications were observed in any group.

## 4 Discussion

This 12‐month real‐world retrospective cohort study demonstrates that ReCell autologous cell suspension was associated with superior overall repigmentation compared with conventional tissue grafting. However, stratified analyses reveal that this advantage is confined to specific anatomical sites and disease subtypes, suggesting that surgical selection should be individualized rather than uniformly applied.

ReCell's key advantage lies in its ability to harvest a small donor graft for uniform application across a recipient area 10 times larger, making it ideal for extensive, multifocal, or anatomically challenging vitiligo [12, 17]. In our stratified analysis by lesion location, for facial/neck/trunk lesions, tissue grafting produced outcomes that were statistically indistinguishable from ReCell at 12 months (*p* = 0.562). This may be attributed to the rich blood supply and adequate melanocyte reserve in these areas, allowing traditional transplanted full‐thickness epidermal sheets to survive well. Traditional grafting techniques offer advantages of shorter operative time (1.5 vs. 2 h) and lower cost in these locations. However, clinicians must counsel patients about the risk of variegated appearance on exposed areas, as documented by Gupta et al. [18].

For acral lesions, neither technique performed well. The repigmentation rates were approximately 50% at best, and the difference between tissue grafting and ReCell was not statistically significant. This suggests that the problem is not the graft itself but the recipient site. Hands and feet have relatively poor vascularity, constant mechanical stress, and a less favorable environment for melanocyte migration. Our data add to the growing recognition that acral vitiligo remains a stubborn challenge across all surgical modalities. Future research can explore more suitable treatment options for acral vitiligo by optimizing surgical protocols, increasing sample size, and designing well‐conducted prospective studies.

At peri‐mucosal sites, ReCell showed a clear benefit. This likely reflects the practical difficulty of securing a solid tissue graft on the mobile, irregular surfaces around the lips, and other mucosal junctions. A liquid cell suspension conforms to these contours more naturally, and the 12‐month data support this mechanical advantage. For patients with peri‐mucosal or similarly complex anatomical involvement, ReCell should be considered the preferred option if available.

The analysis by vitiligo subtype produced another clinically useful distinction. In nonsegmental vitiligo, ReCell was markedly better than tissue grafting. This may be because nonsegmental disease is often multifocal; the ability to cover a large recipient area from a small donor site is a genuine advantage. In segmental vitiligo, the difference disappeared. Both techniques achieved high repigmentation rates, which aligns with previous observations that segmental disease, once stable, tends to respond favorably to surgery regardless of the graft type [17]. This reinforces a key clinical principle: rigorous patient selection—ensuring a true stability before operating—may matter more than the specific tool chosen.

The role of postoperative phototherapy deserves emphasis. Our regression model identified 24–47 sessions of 308‐nm excimer lamp therapy as an independent predictor of success, increasing the odds of a good outcome nearly fourfold. This is consistent with the systematic review by Lommerts et al., which concluded that phototherapy enhances surgical repigmentation by stimulating melanocyte proliferation and melanogenesis [19]. In our practice, not all patients completed phototherapy because of cost, distance, or scheduling constraints. This real‐world variability introduces confounding that we cannot fully resolve with statistical adjustment. Nevertheless, the dose–response pattern is convincing enough that we now routinely recommend postoperative phototherapy twice weekly for at least 3 months. Whether more than 48 sessions add further benefit remains uncertain; our numbers in that category were too small to draw a conclusion.

Safety outcomes also favored ReCell. The overall adverse event rate was low, but hyperpigmentation was noticeably more common with tissue grafting. This probably relates to the uneven distribution of melanocytes when an intact graft is transferred, leaving some areas darker than others. ReCell suspension allows a more even spread, which matters for patients with high aesthetic expectations. That said, none of the techniques produced donor‐site complications in our hands, confirming that all three are safe when performed by experienced operators.

Nevertheless, we must acknowledge that this study has some limitations that cannot be overlooked. As a single‐center retrospective study, it is inevitably subject to selection bias; the uneven distribution of lesion locations and disease types across the two groups was not random; it reflected our own clinical habits—using ReCell for large or complex cases and reserving tissue grafting for smaller or acral lesions. We have tried to address this through stratification and multivariable regression, but residual confounding is inevitable. We did not record precise lesion size or confluence, which almost certainly influenced both technique selection and outcome. Phototherapy and traditional Chinese medicine use were not protocol‐mandated; they varied according to patient preference and economic circumstances. The procedural details necessarily differed between techniques—different dermabrasion targets, different dressings, different anesthesia approaches—so this was a comparison of treatment packages rather than a single‐variable experiment. Unmeasured confounders including individual surgeon‐specific technical proficiency, precise lesion size and confluence, and patient socioeconomic status influencing phototherapy access likely influenced outcomes. Finally, our follow‐up period was only evaluated up to 12 months post‐surgery. Long‐term stability could not be assessed, as repigmentation and recurrence may occur up to 6 years post‐transplantation [20, 21]. Therefore, future research should expand the subgroup sample size or take multicenter randomized controlled trials with standardized adjuvant therapy to verify our results, and extend follow‐up to evaluate long‐term repigmentation rates and color matching.

In conclusion, ReCell offers superior 12‐month repigmentation for stable nonsegmental vitiligo on the face, trunk, and peri‐mucosal sites, while traditional tissue grafting remains a practical, cost‐effective alternative for small facial or trunk lesions. Neither technique produces satisfactory results at acral sites. Adjuvant 308‐nm phototherapy (≥ 24 sessions) is essential for optimizing outcomes regardless of surgical modality. These real‐world findings are hypothesis‐generating; prospective randomized trials are needed to confirm the optimal surgical selection strategy.

## Author Contributions

Shaowei Cheng originally designed the study. The first draft of the manuscript was written by Dihui Lai. The data were assessed by Dihui Lai and Jing Ma. Xueya Tong, Maoying Wei, Xiaoju Xian, and Xintong Chang were responsible for the material preparation and data collection. All authors have made substantial contributions to conception and design. All authors commented on previous versions of the manuscript. All authors read and approved the final manuscript.

## Funding

The authors have nothing to report.

## Conflicts of Interest

The authors declare no conflicts of interest.

## Supporting information


**Figure S1:** Intraoperative technical details of three surgical modalities. (A) Negative pressure suction canister prepared for suction blister induction. (B) Suction heads of varying specifications for different donor area requirements. (C) Donor site harvesting for ultra‐thin split‐thickness skin graft. (D) Dermabrasion of recipient site. (E) Placement of ultra‐thin skin graft onto recipient site. (F) Application of 3M Tegaderm I.V. transparent dressing following ReCell procedure. (G) Sterile gauze placement for compression bandaging.

**Table S1:** Comparison of procedural details the three surgical methods.
**Table S2:** Lesion details of different surgical approaches.
**Table S3:** Stratified analysis of 12‐month repigmentation by anatomical site and vitiligo type.

## Data Availability

The data that support the findings of this study are available from the corresponding author upon reasonable request.
