# Supplementary figures and images for: Comparative Efficacy and Safety of Tissue Grafting Versus ReCell Autologous Cell Suspension in Stable Vitiligo: A 12‐Month Retrospective Study
Source: J Cosmet Dermatol. 2026 Jul 27;25(8):e71086. doi: 10.1111/jocd.71086 (PMC13403090; doi:10.1111/jocd.71086)

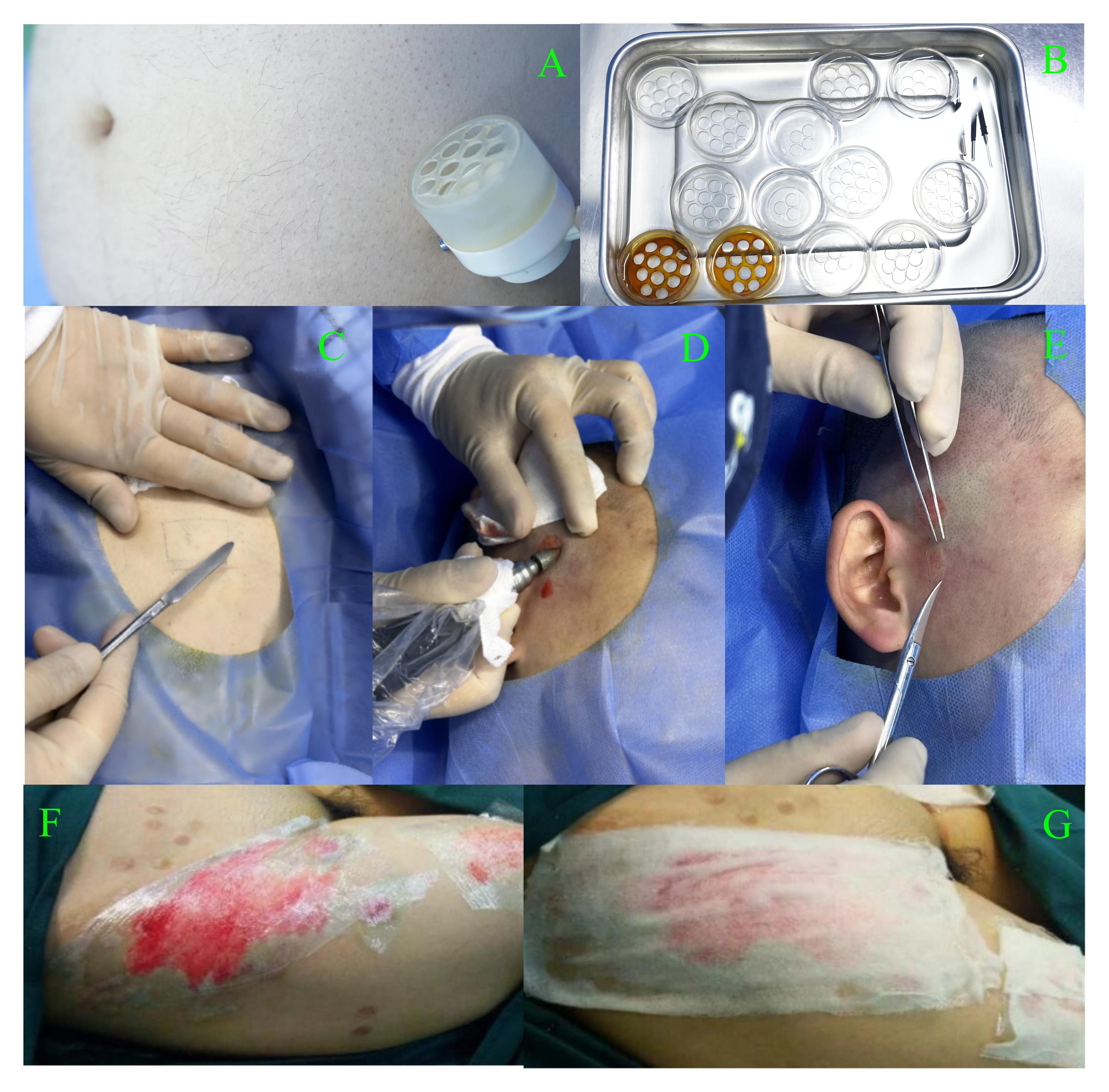

Supplement: Supplementary file 1 — Figure S1: Intraoperative technical details of three surgical modalities. (A) Negative pressure suction canister prepared for suction blister induction. (B) Suction heads of varying specifications for different donor area requirements. (C) Donor site harvesting for ultra‐thin split‐thickness skin graft. (D) Dermabrasion of recipient site. (E) Placement of ultra‐thin skin graft onto recipient site. (F) Application of 3M Tegaderm I.V. transparent dressing following ReCell procedure. (G) Sterile gauze placement for compression bandaging. [file JOCD-25-e71086-s001.jpg]
