# Supplementary material for: Comparative Efficacy and Safety of Tissue Grafting Versus ReCell Autologous Cell Suspension in Stable Vitiligo: A 12‐Month Retrospective Study
Source: J Cosmet Dermatol. 2026 Jul 27;25(8):e71086. doi: 10.1111/jocd.71086 (PMC13403090; doi:10.1111/jocd.71086)
Supplement: Supplementary file 2 — Table S1: Comparison of procedural details the three surgical methods. Table S2: Lesion details of different surgical approaches. Table S3: Stratified analysis of 12‐month repigmentation by anatomical site and vitiligo type. [file JOCD-25-e71086-s002.docx]

**Supplementary Table 1. Comparison of procedural details the three surgical methods**

| **Parameter** | **SBEG** | **UT-STSG** | **ReCell^®^** |
| --- | --- | --- | --- |
| **Donor site anesthesia** | 1% lidocaine without epinephrine, local infiltration | Topical application of Compound Lidocaine Cream | 1% lidocaine without epinephrine, local infiltration |
| **Recipient site anesthesia** | 1% lidocaine without epinephrine, local infiltration | 1% lidocaine without epinephrine, local infiltration | 1% lidocaine without epinephrine, local infiltration |
| **Donor graft thickness** | ＜0.125mm | 0.13mm | 0.15-0.2mm |
| **Routine donor site** | Abdomen | Medial thigh | Medial thigh or perilesional non-affected skin |
| **Recipient site dermabrasion** | Punctate uniform bleeding | Diffuse bleeding | Punctate uniform bleeding |
| **Procedure duration** | 1.5 hours | 1 hour | 2 hours |
| **Donor site postoperative dressing** | 1 layer oil gauze, sterile gauze | Lipidocolloid dressing (Urgotul®, URGO，France) | Lipidocolloid dressing (Urgotul®, URGO，France) |
| **Recipient site postoperative dressing** | 2 layers oil gauze, sterile gauze | Alginate dressing, hydrocolloid dressing, sterile gauze | Tegaderm™ I.V. (3M) transparent dressing, sterile gauze |
| **Donor site postoperative bandage** | Coverage for 1 week | Coverage for 1 week | Coverage for 1 week |
| **Recipient site bandage duration** | Compression bandage for 1 week | Compression bandage, joint immobilization required | Compression bandage for 1 week |
| **Required equipment** | Negative pressure suction device, dermabrasion machine | Blade, dermabrasion machine | ReCell^®^ kit, dermabrasion machine |

SBEG: Suction blister epidermal grafting; UT-STSG：Ultra-thin split-thickness skin grafting

**Supplementary table 2. Lesion details of different surgical approaches**

| **Lesions sites** | **Conventional tissue grafting (451)** | | **Cellular grafting (1050)** | **Total** (1501) | **P** |
| --- | --- | --- | --- | --- | --- |
|  | **SBEG** (336) | **UT-STSG** (115) | **ReCell®** (1050) |  |  |
| Face/neck/trunk | 54(16.07%) | 2(1.74%) | 717(68.29%) | 773(51.50%) | <0.001 |
| Acral | 264(78.57 %) | 92(80 %) | 283(26.95%) | 639(42.57%) |  |
| Peri-mucosal | 18(5.36 %) | 21(18.26%) | 50(4.76%) | 89(5.93%) |  |

**Supplementary table 3. Stratified analysis of 12-month repigmentation by anatomical site and vitiligo type.**

| **Site or type** | **Tissue grafting, n** | **ReCell®, n** | **Median repigmentation rate** | **P** |
| --- | --- | --- | --- | --- |
| Face/neck/trunk | 56 | 717 | 92.86% vs. 98.57% | 0.562 |
| Acral | 356 | 283 | 50.81% vs. 46.94% | 0.077 |
| Peri-mucosal | 39 | 50 | 50.00% vs. 81.03% | 0.028 |
| Nonsegmental | 415 | 867 | 53.86% vs. 87.50% | ＜0.001 |
| Segmental | 15 | 179 | 81.25% vs. 94.96% | 0.374 |
